# Treatment Costs and Social Burden of Pancreatic Cancer

**DOI:** 10.3390/cancers15061911

**Published:** 2023-03-22

**Authors:** Elżbieta Cipora, Olga Partyka, Monika Pajewska, Aleksandra Czerw, Katarzyna Sygit, Marian Sygit, Mateusz Kaczmarski, Dominika Mękal, Edyta Krzych-Fałta, Anna Jurczak, Katarzyna Karakiewicz-Krawczyk, Sylwia Wieder-Huszla, Tomasz Banaś, Ewa Bandurska, Weronika Ciećko, Andrzej Deptała

**Affiliations:** 1 Medical Institute, Jan Grodek State University in Sanok, 38-500 Sanok, Poland; 2 Department of Economic and System Analyses, National Institute of Public Health NIH-National Research Institute, 00-791 Warsaw, Poland; 3 Department of Health Economics and Medical Law, Medical University of Warsaw, 01-445 Warsaw, Poland; 4 Faculty of Health Sciences, Calisia University, 62-800 Kalisz, Poland; 5 Department of Oncology Propaedeutics, Medical University of Warsaw, 01-445 Warsaw, Poland; 6 Department of Basic of Nursing, Faculty of Health Sciences, Medical University of Warsaw, 01-445 Warsaw, Poland; 7 Department of Clinical Nursing, Pomeranian Medical University in Szczecin, 71-210 Szczecin, Poland; 8 Department of Radiotherapy, Maria Sklodowska-Curie Institute-Oncology Centre, 31-115 Cracow, Poland; 9 Center for Competence Development, Integrated Care and e-Health, Medical University of Gdansk, 80-204 Gdansk, Poland

**Keywords:** pancreatic cancer, healthcare costs, indirect costs, economic burden

## Abstract

**Simple Summary:**

According to the forecasts, the share of pancreatic cancer in the structure of gastrointestinal malignancies will increase. This cancer is characterized by high mortality and due to non-specific symptoms, it is mostly diagnosed at an advanced stage of the disease. Late detection and the required highly specialized treatment result in an increased economic burden of the disease. PC generates costs both for the health care system, but also indirect costs resulting mainly from work absence caused by the disease. PC is becoming a significant problem from the perspective of limited system resources and the loss of potential GDP. With the progressive aging of the population, where the demographic structure in developed countries is reversed, it is important to keep as many people of working age in the labor market as possible.

**Abstract:**

(1) Background: Pancreatic cancer is the cancer with the third-highest mortality rate, and forecasts indicate its growing share in morbidity. The basis of treatment is inpatient chemotherapy and there is a strong focus on palliative care. (2) Methods: A literature review was conducted based on the rapid review methodology in PubMed and Cochrane databases. The search was supplemented with publications from the snowball search. Qualitative assessment of included publications was performed using AMSTAR2 modified scheme. (3) Results: The review included 17 publications, of which majority concerned direct costs related to the adopted treatment regimen. Most of the publications focused on comparing the cost-effectiveness of drug therapies and the costs of palliative treatment. Other publications concerned indirect costs generated by pancreatic cancer. They particularly focused on the economic burden of lost productivity due to sickness absence. (4) Conclusion: The increase in the incidence of pancreatic cancer translates into an increase in the costs of the health care system and indirect costs. Due to the significant share of hospitalization in the health care structure, direct costs are increasing. The inpatient treatment regimen and side effects translate into a loss of productivity for patients with pancreatic cancer. Among gastrointestinal cancers, pancreatic cancer generates the second largest indirect costs, although it has a much lower incidence rate than the dominant colorectal cancer. This indicates a significant problem of the economic burden of this cancer.

## 1. Introduction

Gastrointestinal cancers constitute a growing problem among noncommunicable diseases. According to the existing forecasts, the share of pancreatic cancer belonging to this group of diseases, in the incidence structure is increasing. Pancreatic cancer is currently the seventh cause of death due to malignant neoplasm in the world. The identified factors that increase the risk of developing pancreatic cancer include, above all, chronic pancreatitis, radiotherapy of the pancreatic area due to another cancer, diabetes, obesity, smoking and age (the risk of developing the disease increases with age) [1]. In most cases, pancreatic cancer develops asymptomatically. The most common symptoms are non-specific (abdominal pain, nausea, lack of appetite), which complicates and prolongs the diagnostic process [2]. This translates into late detection, usually at an advanced stage. For this reason, curability is low, and the 5-year survival rate is only 9% [3,4]. In addition, patients with pancreatic cancer may be diagnosed with other diseases that should be taken into account during treatment [5].

Chronic diseases, such as cancer, require long-term treatment, are expensive and often limit the social and professional life of patients. They generate two types of costs: direct costs and indirect costs. The first group of costs relates primarily to the health care system’s expenses incurred for medical services and to the patient’s own costs related to treatment, e.g., out-of-pocket expenses for drugs and medical supplies. The second category includes the costs of lost productivity resulting from sickness absence or presenteeism [6]. According to forecasts, the incidence of pancreatic cancer will increase [7], and a larger number of patients will translate into higher treatment costs and a growing loss of productivity due to sickness absence.

The objective of this article is to present the latest available knowledge on the direct and indirect costs of pancreatic cancer as a growing problem for healthcare financing.

## 2. Materials and Methods

In the period from July to September 2022, a literature review was conducted to summarize the state of knowledge about the costs of treatment and the economic burden of pancreatic cancer. The search strategy prepared was based on the MeSH dictionary and Boolean logical operators: “neoplasm” OR “cancer” AND (pancreatic * OR pancreas * OR pancreatic diseases OR pancreatic neoplasms) AND (cost OR costs OR economic analys * OR economic evaluat * OR economic loss OR expenditure * OR spend * OR expense * OR burden OR productivity OR costs and cost analysis) AND (“2017” [DP] OR “2018” [DP] OR “2019” [DP] OR “2020” [DP] OR “2021” [DP] OR “2022” [DP]) Filters: Humans, English” subject to subsequent modifications. The criteria for inclusion of publications according to the PICOS scheme are presented in Table 1.

**Figure 1:**
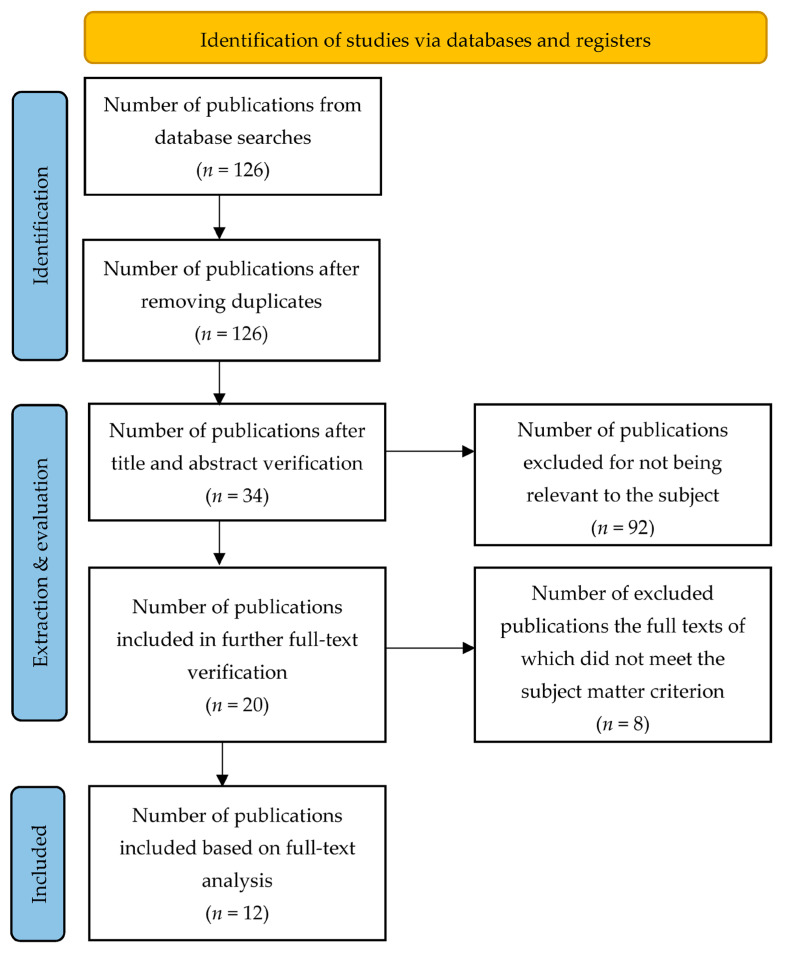
Simplified PRISMA scheme for inclusion of publications for further review.

Based on the elements of the main objectives of the rapid reviews methodology, PubMed and Cochrane databases were searched, narrowing the publication period to the last 6 years. In order to systematize the search, a simplified PRISMA scheme was used (Figure 1).

Then, using snowball sampling, based on the publications included in the review, further items of scientific literature in the subjective scope were added to the review, i.e., *n* = 5. Publications that in the expert opinion of the authors had significant cognitive value were included. In total, 17 publications were included in the review.

Two authors evaluated the abstracts of the publications, qualifying them for further analysis. Potentially eligible publications were then independently reviewed by another two authors. All doubts regarding further inclusion of the publications in the review were resolved by consensus of the entire team of authors.

## 3. Results

Table 2 presents a summary of the publications included in the review with a description of their most important features.

Due to its course and unfavorable prognosis, pancreatic cancer generates significant treatment costs (direct) as well as indirect costs. The severe course of pancreatic cancer is associated with the burden of physical symptoms and side effects of therapy [8]. In patients with pancreatic cancer, a common problem that requires treatment is increased pain caused by many factors, such as compression of the nerves around the pancreas, which causes chronic pain in the lower abdomen and/or back in half of the patients [25]. For this reason, it is necessary to conduct intensive pain therapy, which increases direct costs of treatment. In addition, inadequately conducted pain therapy is associated with limitations in professional activity [26].

### 3.1. Direct Costs

#### 3.1.1. Treatment of Advanced Pancreatic Cancer

Direct costs of treatment of pancreatic cancer are associated primarily with the costs of drugs and the costs of treatment of complications and side effects resulting from treatment [27]. In the study conducted by Peery et al. in the United States in 2014, pancreatic cancer generated over 1.3 million days of hospitalization with an average cost of hospitalization of USD 6240 [9]. Total treatment costs were estimated at USD 2.7 million a year. However, according to Stukalin et al. total expenditure related to healthcare for patients with pancreatic cancer averaged USD 2.55 billion in 2016 [11]. In his publication, Soefje points to the results of economic analyses of direct medical costs conducted on a cohort of patients subjected to oncological treatment in relation to the control group, where PPPM (per-patient-per-month) for patients with pancreatic cancer was on average USD 15,480 to USD 1001 for the control group (the analysis included the costs of hospitalization, outpatient care, visits to the emergency department) [12]. Arciero et al. compared the cost-effectiveness of treatment regimens for patients with advanced pancreatic cancer treated with gemcitabine and nab-paclitaxel (Gem-Nab) or subjected to the FOLFIRINOX regimen (fluorouracil, leucovorin, irinotecan, oxaliplatin). The introduction of new drugs to treat pancreatic cancer allowed to improve the survival rate and quality of life of patients, but it also translated into higher treatment costs. The results of the analysis indicated higher costs of treatment with gemcitabine and nab-paclitaxel. The average five-year cost of treatment was USD 103,884 compared to USD 101,518 for FOLFIRINOX [10]. According to the National Inpatient Sample, between 1997 and 2012, the total cost of pancreatic cancer increased three-fold, from USD 24,000 per hospitalization to USD 68,000, while shortening hospitalization time from 9.6 days to 7.8. In 2017 the National Institute for Health and Care Excellence (NICE) conducted an evaluation of treatment with gemcitabine and nab-paclitaxel using the mixed treatment comparison (MTC) method with a fixed effects model. The results suggested greater clinical effectiveness and cost-effectiveness of this treatment regimen compared to gemcitabine alone, yielding an incremental cost-effectiveness ratio (ICER) of GBP 41,000–46,000 per quality-adjusted year (QALY). In further comparison with the FOLFIRINOX regimen, the results of the evaluation indicated lower clinical effectiveness and cost-effectiveness of gemcitabine and nab-paclitaxel [28]. Similar conclusions were drawn by Coyle et al. in the analysis conducted using the Markov model, where the incremental cost for gemcitabine and nab-paclitaxel was CAD 54,043 with 0.814 life years gained (LYG) and for FOLFIRINOX CAD 26,443 and 1.006 LYD [13]. Also using the Markov model, Gharaibeh et al. found that the FOLFIRINOX regimen shows the highest clinical effectiveness, and treatment with gemcitabine and nab-paclitaxel was the most expensive [15]. However, in the study conducted by Bullock et al. for the costs of healthcare for patients with metastatic adenocarcinoma of the pancreatic duct who started first-line chemotherapy, the costs of the FOLFIRINOX regimen were higher than the costs of nab-paclitaxel with gemcitabine [16]. Up to six months after the index date, the median of total cumulative healthcare costs per patient was USD 85,714 for nab-paclitaxel with gemcitabine and USD 114,116 for FOLFIRINOX. Up to twelve months from the index date, the median was USD 144,264 for nab-paclitaxel with gemcitabine and USD 203,224 for FOLFIRINOX. The main differences in costs in these treatment regimens for metastatic adenocarcinoma of the pancreatic duct were observed in outpatient healthcare (procedures, drugs, diagnostic tests, health services), which were lower for gemcitabine and nab-paclitaxel therapy, while no differences in costs were observed in inpatient treatment. Similar conclusions were drawn from the retrospective study conducted by McBride et al. as nab-paclitaxel with gemcitabine, as a first-line drug, was associated with lower costs of pharmacy supplies, administration procedures and supportive care. Due to significant methodological differences and a large number of confounding variables, further research on this topic is required [17]. In the study conducted by Malangone-Monaco et al., mean costs per patient per month of treatment (PPPM) for first-line FOLFIRINOX treatment was estimated at USD 29,526, of which USD 7138 was the cost of inpatient treatment and USD 22,388 was the cost of outpatient treatment. For the gemcitabine and nab-paclitaxel regimen, the total PPPM was USD 27,403, inpatient treatment was estimated at USD 8993 and outpatient treatment at USD 18,410 [14].

Due to the differences in methodology adopted by the authors, it is not possible to unanimously indicate the treatment regimen that would be the most cost-effective. The decision to implement the selected treatment is influenced by many factors, and the progress of science provides new therapeutic options that tend to show greater clinical and cost effectiveness. In the discussed studies, depending on the situation, the FILFIRINOX regimen was more cost-effective than the nab-paclitaxel with gemcitabine, but in the remaining studies the situation was reversed.

#### 3.1.2. Adjuvant and Neoadjuvant Treatment

In the case of radical treatment of pancreatic cancer, adjuvant treatment with chemotherapy or combined with radiotherapy is often included. Adjuvant treatment is implemented after resection, while neoadjuvant treatment is carried out before surgery. In the study conducted by Arjani et al. cost-effectiveness analysis of the surgical removal of pancreatic cancer and neoadjuvant treatment in patients with resectable cancer was performed. The results indicated greater cost-effectiveness of neoadjuvant treatment with subsequent surgical treatment. It was USD 6840 more expensive than resection combined with adjuvant treatment, but showed a 0.14 higher QALY. The ICER was estimated at USD 48,130/QALY [24]. In another study on healthcare costs, Cerullo et al. attempted to assess the financial burden of various therapeutic options available in the USA for resectable pancreatic cancer. The median of the tumor resection costs ranged from USD 37,030 for distal pancreatectomy to USD 57,893 for Whipple pancreatoduodenectomy. The median of the cumulative chemotherapy costs for all patients was USD 39,098, while the costs of gemcitabine treatment were estimated at USD 39,041 and in combination with nab-paclitaxel, the costs almost doubled to USD 74,051. A comparable cost was obtained for the FOLFIRINOX therapy, i.e., USD 70,419. Large differences in costs were also observed for radiotherapy. For patients who received radiotherapy after being subjected to the FOLFIRINOX regimen, the median of the total cost was USD 93,336, while for patients treated with radiotherapy with oral capecitabine, the total cost was USD 19,294. The median of the cumulative cost for gemcitabine and radiotherapy was estimated at USD 46,074. In pancreatic cancer, the financial burden of each type of treatment varies significantly depending on the period in which this type of analysis was performed, as it is related to the progress in adjuvant treatment resulting from the use of more modern chemotherapy regimens and combination procedures (e.g., chemotherapy + radiotherapy).

Therefore, the costs of chemotherapy and radiotherapy after resection, as well as subsequent hospitalization, varied significantly between patients. The estimated total treatment costs were the highest among patients receiving FOLFIRINOX or gemcitabine and nab-paclitaxel. In contrast, radiotherapy was associated with cumulative costs in the range of USD 20,000–90,000 depending on the chemotherapy received. Including tumor resection costs, the median of the total cost of oncological care was over USD 150,000 for patients who received neoadjuvant and adjuvant treatment. These results highlight significant expenses associated not only with surgery, but also with the overall multimodal treatment plan often used in the care of patients with pancreatic cancer. According to available knowledge, further research is needed on the cost-effectiveness of various therapies, also taking into account factors such as quality of life or expenses directly incurred by the patient in connection with the therapy administered. Quality of life, which plays a large role in palliative treatment, is particularly important in cancer with a difficult prognosis, such as pancreatic cancer [18].

#### 3.1.3. Supportive and Palliative Care

Due to the low survival rates in metastatic pancreatic cancer, it is important to take into account the costs of adjuvant care when assessing healthcare costs. This care is part of integrated palliative and supportive cancer care. Guidelines for adjuvant care are included in the European Code of Cancer Practice [29]. The goal of supportive care in patients with advanced cancer is to achieve a balance between positive therapeutic results and side effects, i.e., ensuring optimal quality of life during treatment. Kang et al. attempted to estimate the medical costs of supportive care in terminal patients. Patients with pancreatic cancer were hospitalized more often than others, which resulted in higher system costs. The average cost of all medical supportive care services was USD 7702, and the hospitalization costs were USD 7131. The mean per-patient-per-month (PPPM) cost for pancreatic cancer was USD 3156, and it was the second highest cost per month for supportive care for lung cancer. Among all cancers analyzed by the authors, pancreatic cancer was characterized by the highest rate of hospitalization and the longest hospitalization period. In addition, due to the severity of pain, it required the administration of strong opioid analgesics. All this translated into higher total costs of care for a patient with pancreatic cancer compared to other cancers. Analyzing healthcare expenditures on oncological care on the example of the United States, pancreatic cancer is one of the most expensive gastrointestinal cancers. According to the cost projection, in 2016 pancreatic cancer was the second, after colorectal cancer, cancer with the highest systemic cost—USD 2.55 billion. Hospital care had the largest share in the structure of direct costs (64.5% of costs) [19].

The overall high costs of pancreatic cancer treatment result to a large extent from the difficulties in diagnosing this cancer and its clinical image. Even in highly developed countries, the disease is diagnosed when it reaches the metastatic or locally advanced stage, which excludes surgical treatment and forces patients to undergo chemotherapy or chemotherapy combined with radiotherapy. Due to the lack of sensitive and specific tumor markers and the lack of a generally accepted standard of screening for pancreatic cancer, early detection of the disease is rare. Current screening, often with high-cost diagnostics such as magnetic resonance imaging (MRI) or endoscopic ultrasonography (EUS), is limited to people with a family history of pancreatic cancer or genetic risk factors (BRCA2 mutation, Lynch syndrome, or familial adenomatous polyposis) [30]. According to the analyses of Corral et al. conducted for the US population, cost-effectiveness of these two examinations was dependent on the patients’ age, where the use of MRI was more effective in the 40–70 age group, and as the cost of MRI increased above USD 1600, endoscopic ultrasound became more cost-effective [20].

### 3.2. Indirect Costs

In addition to direct costs of healthcare, attention should also be paid to indirect costs of pancreatic cancer. According to the Swedish study conducted by Draus et al. for 2018, the cost of treating one patient was calculated at EUR 16,066, while the cost of lost productivity was almost seventeen times higher, and amounted to EUR 287,420 per working person with pancreatic cancer. Total costs of pancreatic cancer were estimated at EUR 124 million, of which direct costs accounted for EUR 26 million, or 21% of the total costs. Indirect costs amounted to EUR 99 million, accounting for 79% of all costs. The highest costs were generated by people aged 50–64, i.e., 51% of costs. For people of working age, under 65, the overall indirect costs amounted to EUR 328,344 per person. According to the projection developed using a linear regression model, indirect costs will amount to EUR 190 million and will account for 84% of the total costs of the economic burden of pancreatic cancer. For people under 65, indirect costs per person were estimated at EUR 380,738. The second cost forecast based on average incidence rates assumes an increase in costs to EUR 210 million in 2030, of which indirect costs will account for 85% of the total amount of EUR 179 million. For people of working age, the cost of lost productivity due to the disease can be EUR 382,109. The difference in the percentage of indirect costs should be explained by the change in the age distribution resulting from the maturity calculation. The loss of workforce in the group of patients of working age constitutes the largest share in the total costs caused by pancreatic cancer. The change in the incidence of pancreatic cancer in this group has a greater impact on the economic outcome than the change in the incidence in the group of retirees. Both forecasts indicate a high social burden with costs of pancreatic cancer [21]. In the systematic review conducted by Hernandez et al., the average indirect cost per patient was EUR 154,257 or EUR 14,568 PPPM [22]. The report of the Swedish Institute for Health Economics on the costs of gastrointestinal cancers in Europe shows that the indirect costs of pancreatic cancer are the second highest among cancers (EUR 3.3 billion), followed only by colorectal cancer (EUR 3.7 billion) [23]. In comparisons between European countries, the highest indirect costs of pancreatic cancer per capita are visible in the richest EU countries, such as Switzerland, Denmark or Germany. The lowest costs are recorded in Bulgaria and Romania. Cross-country differences in costs are mainly a reflection of differences in earnings. This means that the patient’s loss of earnings due to the disease is higher in richer countries. In fact, richer European countries tend to have fewer potential working life years lost because of cancer due to higher survival rates in these countries [23].

In addition to the costs resulting from the loss of patient productivity, an important issue in assessing the indirect costs of pancreatic cancer is the reduction in the workforce of caregivers of patients. The development of treatment methods allows to extend the survival time of patients, but contributes to the economic burden of informal caregivers, e.g., families. Some studies have shown that the family income of a person suffering from pancreatic cancer is lower than before the disease, and the annual expenditure on cancer treatment is 1.18 times the average annual family expenditure [31]. This is consistent with previous results from the Pancreatic Cancer Action Network, which showed that 40% of caregivers, mostly women with an average age of 46.8 years, had to give up work to care for a person with pancreatic cancer [32].

Higher indirect costs probably result from the specific course of pancreatic cancer, where most cases are diagnosed at an advanced stage and consequently have a lower survival rate. Patients who live longer may receive treatment for a longer time, which increases direct costs. Higher survival rates mean lower death rates among cancer patients. Fewer deaths mean less productivity loss due to mortality, but potentially also higher productivity loss due to morbidity.

## 4. Limitations

This article is not a systematic review, but in order to increase its substantive value, the authors based their research on the methodology of systematic reviews. This article is one of only a few publications attempting to collect the most important information on healthcare costs, economic burden and especially indirect costs, which are often overlooked in economic analyses of cancer treatment. The comparison of results is a complicated task due to differences in cost calculation methodology, but also due to differences in the organization and financing of services at the level of healthcare systems in individual countries, which is why the authors limited themselves to presenting the latest and relevant information available on the economic effects of pancreatic cancer without performing detailed comparative analysis. The economic effects of targeted treatment in metastatic pancreatic cancer were also not evaluated (PARP inhibitors in subtypes with *BRCA1/BRCA2* gene mutations, anti-PD-1/PD-L1 immunotherapy in subtypes with high microsatellite instability, RAS pathway inhibitors in subtypes with *KRAS- G12C* mutation, etc.) due to the lack of high-quality cost-consumption analyses.

## 5. Conclusions

Forecasts indicate a growing share of pancreatic cancer in the structure of neoplastic diseases. Due to diagnostic difficulties and poor prognosis in treatment and survival, pancreatic cancer will constitute an increasing financial challenge for the healthcare system. Due to the significant share of hospitalization in the healthcare for patients with pancreatic cancer, the direct costs of healthcare increase. An additional increase in costs is also caused by the requirements of palliative treatment, in particular pain therapy, as pain is a common problem in pancreatic cancer. The inpatient treatment regimen and side effects translate into a loss of productivity for patients with pancreatic cancer. Among gastrointestinal cancers, pancreatic cancer generates the second highest indirect costs, although it has a much lower incidence rate than colorectal cancer. This shows a significant economic burden of this cancer. With the increase in the number of cases of pancreatic cancer, direct and indirect costs will increase, becoming a significant problem from the perspective of limited system resources and the loss of potential GDP as a result of long sickness absence or death. With the progressive aging of the population, where the demographic structure in developed countries is reversed, it is important to keep as many people of working age in the labor market as possible.

## Figures and Tables

**Table 1:** Criteria for inclusion of publications according to the PICO scheme.

**Population (P)**	Patients Diagnosed with Pancreatic Cancer
**Intervention (I)**	Economic analyses focused on the costs of pancreatic cancer treatment, assessment of the economic burden of indirect costs of pancreatic cancer
**Comparator (C)**	Any or none
**Outcomes (O)**	Direct costs of pancreatic cancer treatment, indirect costs of pancreatic cancer, economic burden
**Studies (S)**	Case studies, prospective studies, retrospective studies, systematic review, RCT
**Limitations**	Publications in English assessing the impact of pancreatic cancer on the quality of life, publication period 2017–2022
**Exclusion**	Non-English publications, studies not directly linked to pancreatic cancer

**Table 2:** Characteristics of publications included in the review.

Author/Year	Country	Unit of Measure	Methodology	Type of Costs	Group of Patients
Carrato A. et al., 2015 [8]	European Union	QALY, EUR	Systematic review	Direct costs, indirect costs	Patients with pancreatic cancer
Peery A. et al., 2018 [9]	USA	USD	Retrospective cohort study	Direct costs	Patients with pancreatic cancer
Arciero V. et al., 2022 [10]	Canada	ICER, USD, INMB	Systematic review	Direct costs	Patients with advanced pancreatic cancer >18 years of age receiving first-line treatment with gemcitabine, nab-paclitaxel, irinotecan, oxaliplatin, in the period from 17 April 2015 to 31 March 2019 registered in the database Cancer Care Ontario’s New Drug Funding Program
Stukalin I. et al., 2022 [11]	USA	USD	Retrospective observational study	Direct costs	Patients with pancreatic cancer
Soefje S.A., 2019 [12]	USA	USD	Retrospective cohort study	Direct costs	Patients with pancreatic cancer
Coyle D. et al., 2017 [13]	Canada	USD, QALY	Cost-effectiveness analysis	Direct costs	Patients receiving first-line treatment for advanced pancreatic cancer or adenocarcinoma
Malangone-Monaco E. et al., 2020 [14]	USA	USD	Retrospective cohort study	Direct costs	6360 patients with metastatic pancreatic cancer (mPC), without secondary diagnosis, not subjected to oncological treatment before mPC diagnosis
Gharaibeh M. et al., 2018 [15]	United Kingdom	QALY, GBP, ICUR	Cost-effectiveness analysis	Direct costs	Phase III clinical trial patients, mainly adults diagnosed with metastatic or advanced pancreatic ductal adenocarcinoma without prior chemotherapy
Bullock A. et al., 2020 [16]	USA	USD	Retrospective cohort study	Direct costs	Cohort of 2199 patients (1352 treated with nab-paclitaxel plus gemcitabine and 847 with FOLFIRINOX)
McBride A. et al., 2017 [17]	USA	USD	Retrospective cohort study	Direct costs	Patients with at least two medical claims related to pancreatic cancer and at least one medical claim related to secondary malignancy on or after the first diagnosis of pancreatic cancer in the period from 1 April 2013 to 31 March 2015
Cerullo M. et al., 2018 [18]	USA	USD	Retrospective cohort study	Direct costs	Patients >18 years of age with a primary diagnosis of pancreatic cancer who have undergone distal or total pancreatectomy or pancreatoduodenectomy.
Kang D-W. et al., 2022 [19]	South Korea	USD	Retrospective cohort study	Direct costs	Patients with pancreatic cancer treated with anticancer drugs during the period from 1 January 2006 to 30 June 2015. The period of best supportive care was defined as the time from the date of taking the last anticancer drug to death.
Corral J. et al., 2019 [20]	USA	USD, ICER	Cost-effectiveness analysis	Direct costs	Hypothetical cohort of 10,000 high-risk individuals (RR = 5-fold) as recommended by the consortium Cancer of the Pancreas Screening (CAPS)
Draus T. et al., 2021 [21]	Sweden	EUR	Retrospective cohort study	Indirect costs	Patients with pancreatic cancer broken down by gender and age group representative for Sweden
Hernandez D. et al., 2022 [22]	European Union, Iceland, Norway, Switzerland, United Kingdom	EUR	Systematic review	Indirect costs	Patients with pancreatic cancer
Hofmarcher T., Lindgren P., 2020 [23]	European Union, Iceland, Norway, Switzerland, United Kingdom	EUR	Assessment of the cost of the disease	Direct costs, indirect costs	Patients with pancreatic cancer
Arjani S. et al., 2022 [24]	USA	USD, ICER	Cost-effectiveness analysis	Direct costs	Two cohorts of patients with pancreatic cancer. The first group was treated surgically, the second group received neoadjuvant FOLFIRINOX therapy and radiotherapy

ICER—incremental cost-effectiveness ratio; QALY—quality-adjusted life year; ICUR—incremental cost-utility ratio; INMB—incremental net monetary benefit.

## Data Availability

Data available on request at the authors.
